# Ribosome-Inactivating Protein α-Momorcharin Derived from Edible Plant *Momordica charantia* Induces Inflammatory Responses by Activating the NF-kappaB and JNK Pathways

**DOI:** 10.3390/toxins11120694

**Published:** 2019-11-26

**Authors:** Ying-Jie Chen, Jia-Qian Zhu, Xiu-Qiong Fu, Tao Su, Ting Li, Hui Guo, Pei-Li Zhu, Sally Kin-Wah Lee, Hua Yu, Anfernee Kai-Wing Tse, Zhi-Ling Yu

**Affiliations:** 1 Center for Cancer and Inflammation Research, School of Chinese Medicine, Hong Kong Baptist University, Kowloon Tong, Hong Kong, China; 2 State Key Laboratory of Quality Research in Chinese Medicine, Institute of Chinese Medical Sciences, University of Macau, Macao, China; 3 Food Science and Technology Program, Beijing Normal University-Hong Kong Baptist University United International College, Zhuhai 519087, China

**Keywords:** alpha-momorcharin, inflammation, JNK, NF-kappaB, ribosome inactivating protein

## Abstract

Alpha-momorcharin (α-MMC), a member of the ribosome-inactivating protein (RIP) family, has been found in the seeds of *Momordica charantia* (bitter melon). α-MMC contributes a number of pharmacological activities; however, its inflammatory properties have not been well studied. Here, we aim to determine the inflammatory responses induced by recombinant α-MMC and identify the underlying mechanisms using cell culture and animal models. Recombinant α-MMC was generated in Rosetta™(DE3)pLysS and purified by the way of nitrilotriacetic acid (NTA) chromatography. Treatment of recombinant α-MMC at 40 μg/mL exerted sub-lethal cytotoxic effect on THP-1 monocytic cells. Transcriptional profiling revealed that various genes coding for cytokines and other proinflammatory proteins were upregulated upon recombinant α-MMC treatment in THP-1 cells, including MCP-1, IL-8, IL-1β, and TNF-α. Recombinant α-MMC was shown to activate IKK/NF-κB and JNK pathways and the α-MMC-induced inflammatory gene expression could be blocked by IKKβ and JNK inhibitors. Furthermore, murine inflammatory models further demonstrated that α-MMC induced inflammatory responses in vivo. We conclude that α-MMC stimulates inflammatory responses in human monocytes by activating of IKK/NF-κB and JNK pathways, raising the possibility that consumption of α-MMC-containing food may lead to inflammatory-related diseases.

## 1. Introduction

Ribosome-inactivating proteins (RIPs) are highly potent protein toxins which restrain protein synthesis by directly targeting the ribosomes [1]. Plant-derived RIPs can be classified into two main types: (1) a polypeptide chain of about 30 kDa (Type I) and (2) a more toxic heterodimer composed of an A chain which has identical function to the Type I RIP, attached with a B subunit that can bind to lectin (Type II) [1]. RIPs can penetrate to the cell by firstly binding to membrane receptors, then passing through the cell membrane by endocytosis and eventually passing in the cytoplasm through shifting from endocytic compartments [2]. Recently, RIPs gain biologists’ attention in the biomedical research area because of their diverse activities toward plant and animal cells, including their anticancer, antiviral, antifungal, abortifacient, and antiparasitic effects [2,3,4,5].

Up to now, the mechanism of RIP-induced biological activities remains unclear, and in some cases the activities are independent from their protein synthesis inhibitory ability. Nowadays, RIP-related biotechnological research is focused on obtaining a deeper understanding of the mechanism of cell entry and also finding ways to increase specificity, reduce antigenicity, and prolong plasma half-life [1]. These unsolved factors strongly hamper the clinical use of RIPs, and are rigorously under investigation. Another factor that limits the clinical use of RIPs is their inflammatory-inducing abilities, in which many RIPs can induce cytokine production and trigger inflammatory responses [6]. For example, RIPs including viscumin [7], ricin [8], Shiga toxins [9], modeccin [10] and eutirucallin [11] can induce cytokines production like tumor necrosis factor (TNF) and interleukins in macrophages or monocytes. Consistent with the cellular observations, TNF and other cytokines levels were upregulated in serum of mice [12] and rats [8] upon RIP ricin treatment. Interleukins levels were also induced by trichosanthin (a Type I RIP) in both peritoneal macrophages [13] and in animals [14]. The inflammatory responses induced by RIPs hamper its clinical use. For example, RIP viscumin from Common Mistletoe (*Viscum album*) and riproximin from *Ximenia americana* exerted therapeutic effects in cancer patients by inhibiting the cancer cell growth; however, it also caused activation of the immune system and the induction of cytokines in immune cells in patients and volunteers taking mistletoe extracts [9,15]. Up to now, the mechanism of cytokines induction by RIPs is not fully understood. The inflammatory-inducing mechanisms of RIPs include the activation of protein kinases such as JNK, p38, and MAPK [12] and key inflammatory-regulating transcription factors (NF-κB, AP-1, etc.) [16].

RIPs are widespread in the plants and distributed in different parts of plant tissues (seed, leaf, sarcocarp, bark) and lattices [6]. RIPs can be found in edible plants, in which some of them are consumed raw by humans [17]. RIPs may undergo degradation under high cooking temperature but RIPs in some plant tissues such as *Spinacia oleracea*, *Daucus carota*, *Cucurbita moschata*, *Allium coepa*, the seeds of *Lycopersicon esculentum* or *Apium graveolens* are actually eaten raw [17]. Furthermore, the leaves of spinach in which the presence of RIP was reported, are frequently appended to uncooked salads [18]. Moreover, the powdered form of the seeds of *Momordica charantia*, which contains RIPs such as α- and β-momorcharin (MMC) [19,20], are commonly employed as granule by Chinese medical doctors and public for relieving high blood pressure and cholesterol level and also for lowering the blood glucose. α-MMC belongs to Type I RIP which exerts various biological activities including RNA hydrolase activity, RNA N-glycosidase activity, protein synthesis inhibitory property, DNA hydrolase, and anti-cancer activity [21,22,23,24].

Many RIPs have been reported to induce pro-inflammatory cytokine production [7,8,9,10,11,12,13,14] but the effects of α-MMC on the immune responses are still not known. Various immune-related adverse effects on the endocrine, gastrointestinal, genitourinary, hematologic, and hepatic systems have been reported due to the consumption of *Momordica charantia* [19]. However, no comprehensive studies have been undertaken to investigate its immune-related mechanisms and also the potential adverse effects of taking it as nutritional supplement. In this study, we propose to carry out a detailed preclinical study to determine the inflammatory responses induced by recombinant α-MMC using cell culture and animal models. Additionally, we sought to define the underlying molecular mechanisms of how α-MMC can induce cytokine production.

## 2. Results

### 2.1. Heterologous Expression and Cytotoxicity of the Recombinant α-MMC

**Figure 1:**
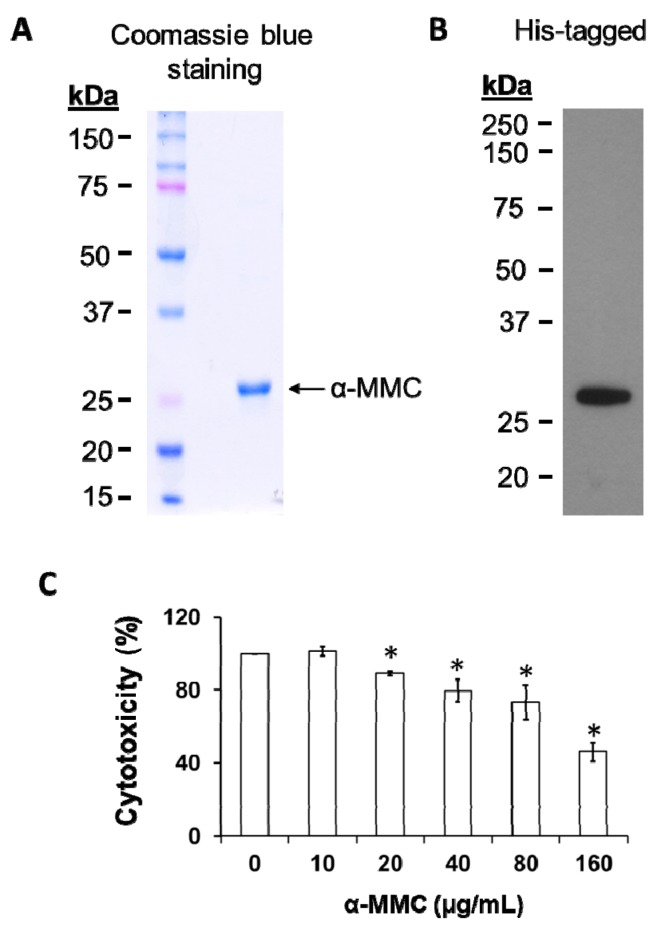
Synthesis of recombinant alpha-momorcharin (α-MMC). (**A**) SDS-PAGE of purified recombinant α-MMC visualized by Coomassie blue staining. (**B**) Western blot analysis of purified recombinant α-MMC protein using anti-6×his-tagged antibody. (**C**) THP-1 cells were untreated or treated with different amounts of α-MMC (0–160 μg/mL) for 24 h. Viability of cells was assessed by 3-(4,5-Dimethyl-2-thiazolyl)-2,5-diphenyl-2H-tetrazolium bromide (MTT) cytotoxic assay. The data are shown as the mean ± SD of three replicates. Significant differences: * *p* < 0.05 compared to control.

We successfully cloned, expressed, and purified recombinant α-MMC from *Escherichia coli* host strains Rosetta (DE3) pLysS for the cell culture and animal studies proposed in this project. The isolation of recombinant His-tagged α-MMC protein was achieved by Ni-NTA affinity chromatography and the purity was shown in 12% SDS-PAGE electrophoresis (Figure 1A). In our expression system, approximate 50 mg recombinant protein could be purified from 1 L of Rosetta culture. The presence of recombinant α-MMC was confirmed by detection of a specific band at nearly 29 kDa with Western blot analysis using anti-6×histidine antibody (Figure 1B). Cell viability was not significantly changed at 24 h treatment time interval by recombinant α-MMC at a concentration of up to 40 µg/mL (<20% growth inhibitory effect) but significantly caused cell death at 160 µg/mL (Figure 1C). α-MMC at a dosage of 40 µg/mL (~IC20) was employed in the following inflammation experiments in vitro.

### 2.2. Microarray Analyses of α-MMC-Induced Inflammatory Responses

RIPs have been reported to trigger inflammation in lymphoid and intestinal organs and also stimulate blood mononuclear cells to produce inflammatory cytokines [2]. Moreover, α-MMC has been found to exert immune-responses in vivo [20,25]. To investigate the expression of inflammatory mediators, human THP-1 monocytic cells were incubated with 40 µg/mL of recombinant α-MMC or 1 µg/mL LPS (sub-lethal dose) as positive control for 24 h, and then gene expression analysis was performed using the Human Inflammatory Response and Autoimmunity RT^2^ Profiler™ PCR Array (Qiagen, CA, USA). We made use of this array to measure the expression pattern of 84 inflammatory genes in each sample. In comparison to untreated and lipopolysaccharides-treated samples, the differentially regulated transcripts with fold changes >2 in α-MMC-treated sample are shown in Table 1. Various inflammatory responses transcripts were significantly upregulated after 24 h incubation with α-MMC and LPS including interleukin 1 beta (IL-1β), chemokine (C-X-C motif) ligand 8 (IL-8), chemokine (C-C motif) ligand 2 (MCP-1), and tumor necrosis factor alpha (TNF-α), etc. For most regulated inflammatory genes, the fold changes of transcripts were higher in LPS-treated sample compared with α-MMC-treated sample including interleukin 6 (IL-6), chemokine (C-C motif) ligands 4 (*CCL4*), etc. For some inflammatory genes, i.e., Fas ligand (*FASLG*) and interferon gamma (*IFNG*), the fold changes of transcripts were higher in α-MMC-treated sample compared with LPS treatment. Overall, these results indicated that α-MMC was able to exert inflammatory responses in human immune cells through LPS/TLR4-dependent or -independent molecular pathways [26].

### 2.3. Recombinant α-MMC Induced Cytokine mRNAs Production and Cytokine Secretion in THP-1 Cells

**Figure 2:**
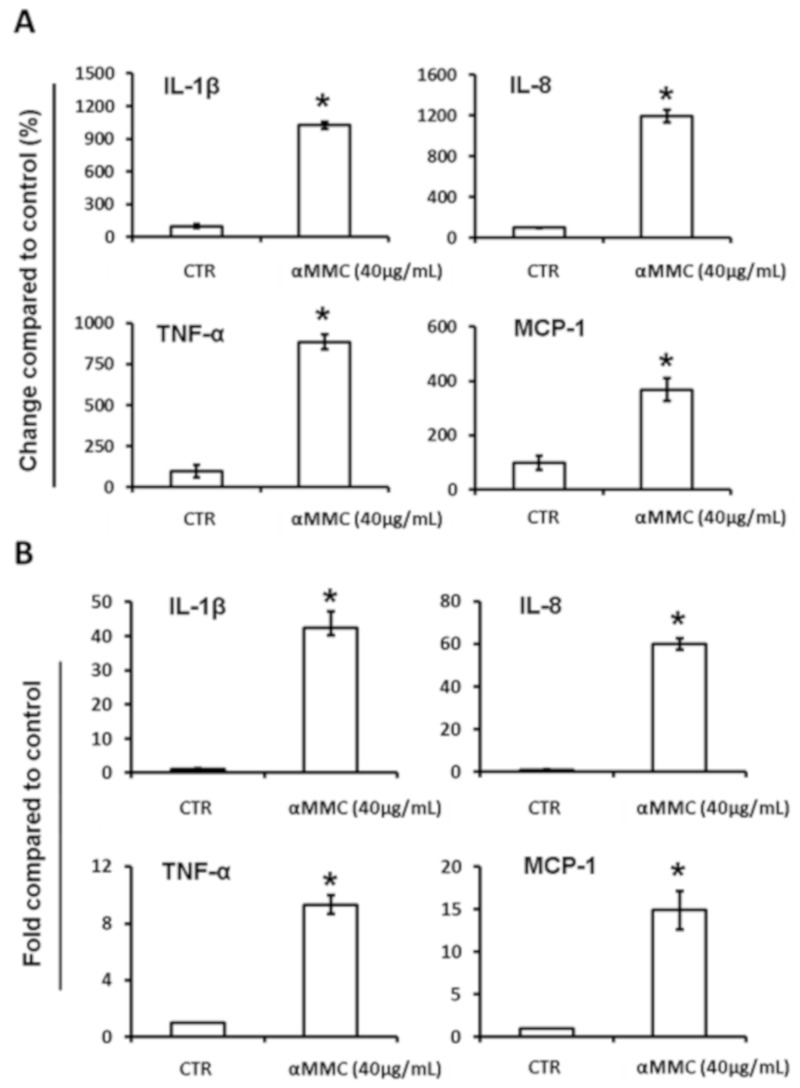
Effect of α-MMC on the pro-inflammatory cytokines’ gene expression and secretion in cultured THP-1 cells. THP-1 cells were incubated with vehicle control or α-MMC (40 μg/mL) for 24 h at 37 °C and then (**A**) the levels of indicated cytokines in THP-1 cell culture medium were detected by ELISA. The results are expressed as percentage of change compared to control group. The data represent the mean ± SD of three replicates. Significant differences: * *p* < 0.05 compared to control. (**B**) IL-1β, IL-8, TNF-α, and MCP-1 mRNA levels were analyzed by RT-PCR. Results are shown as fold-change compared to the control. The data represent the mean ± SD of three replicates. Significant differences: * *p* < 0.01 compared to control.

To confirm the inflammatory-inducing effects by α-MMC, we further tested the expression of several cytokines in α-MMC-treated THP-1 cells. We firstly determined whether α-MMC would induce the major cytokine production and secretion into the medium using human cytokines ELISA kits (eBioscience, San Diego, CA, USA). As shown in Figure 2A, α-MMC induced all four major cytokines’ secretion (IL-1β, IL-8, TNF-α, and MCP-1) in THP-1 cells. As indicated in Figure 2B, IL-1β, IL-8, TNF-α, and MCP-1 mRNA levels were also induced by α-MMC with 3–12-fold increase and these effects were comparable to cytokine secretion. These results indicate that α-MMC exerted a similar cytokine mRNAs and secretion induction pattern compared with other immune-inducing RIPs, which suggests that α-MMC is an immune-modulator.

### 2.4. α-MMC-Induced Inflammatory Responses through NF-kappaB Pathway

**Figure 3:**
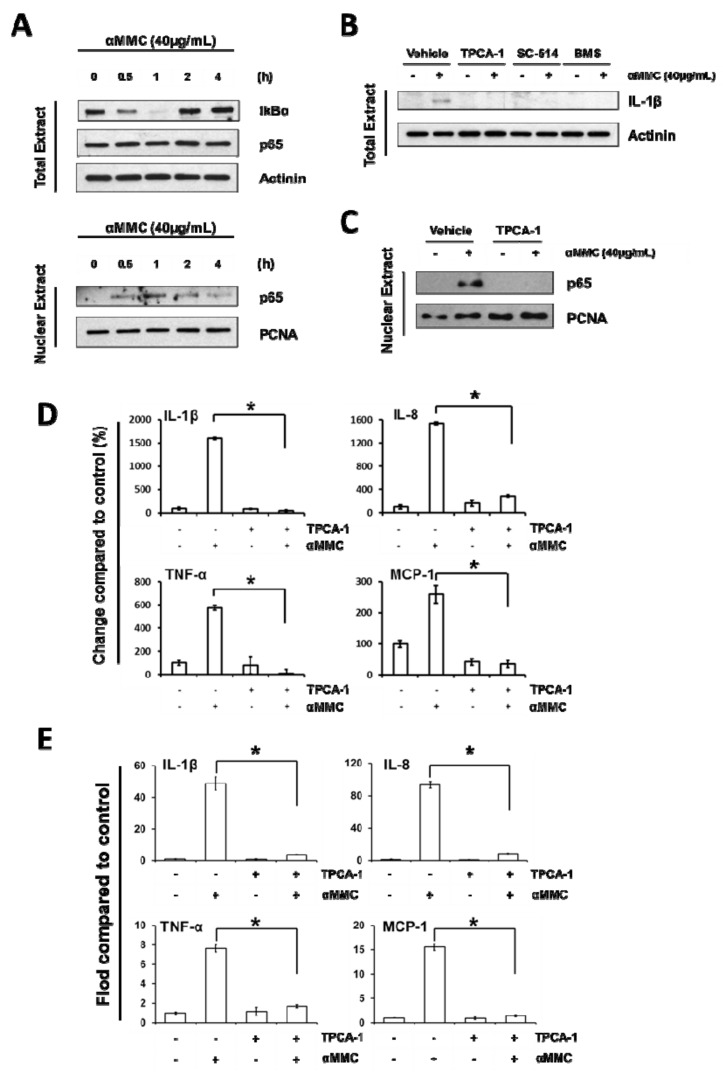
NF-κB activation is essential in α-MMC-induced inflammatory responses in THP-1 cells. (**A**) THP-1 cells were incubated with α-MMC (40 μg/mL) for various time intervals (0, 0.5, 1, 2, 4 h) and levels of IκBα and p65 proteins in the whole cell lysates and p65 protein in the nucleus were assayed by Western blot. Actinin and PCNA were employed as loading control for whole cell and nuclear extracts, respectively. (**B**) THP-1 cells were pretreated with specific IKKβ inhibitors TPCA-1 (5 µM), SC-514 (50 µM), BMS-345541 (2.5 µM) for 30 min, and then the cells were further treated with vehicle control or α-MMC (40 μg/mL) for 24 h. Western blotting was used to analyze the protein expression levels of IL-1β in whole-cell extracts. Actinin was employed as loading control. (**C**) THP-1 cells were exposed to vehicle or α-MMC (40 μg/mL) for 24 h after pretreatments with or without 5 μM TPCA-1 for 30 min. Western blot analysis of nuclei p65 is shown. PCNA was employed as loading control of nuclear extracts. (**D**) THP-1 cell cultures were pretreated with specific IKKβ inhibitor TPCA-1 (5 μM) for 30 min, follow by the addition of α-MMC (40 μg/mL) and further incubated for 24 h. The secretion levels of indicated cytokines in the cell culture supernatant were examined using corresponding ELISA kits. The results are expressed as the change in percentage compared to vehicle control group. The data represent the mean ± SD of three replicates. Significant differences: * *p* < 0.01. (**E**) THP-1 cells were incubated with specific IKKβ inhibitor 5 μM TPCA-1 for 30 min prior to the treatment of 24 h with α-MMC (40 μg/mL). The mRNA levels of the four pro-inflammatory cytokines were analyzed by RT-PCR. Results were expressed as fold-change compared to the vehicle control group. The data represent the mean ± SD of three replicates. Significant differences: * *p* < 0.01.

Activation of IKK-NF-κB is a crucial step participated in the induction of pro-inflammatory cytokines under external or intracellular stimuli [16]. Recent studies revealed that ribosome-inactivating protein and related proteins can activate the IKK-NF-κB pathway [27,28], which lead us to postulate that the IKK-NF-κB pathway may contribute to α-MMC triggered cytokine induction in THP-1 cells. To verify this hypothesis, THP-1 cells were harvested at various time intervals after the treatment of α-MMC, and the whole and nuclear cell lysates were then extracted followed by testing the NF-κB activity using Western blot analysis. Our results show that α-MMC induced a quick IκBα degradation at 60 min, following with a gradual IkBα restoration at 120 min (Figure 3A). Since transcriptional activity of NF-κB is dependent on a p65 subunit [14], we determined the level of p65 inside the nucleus. As shown in Figure 3A, α-MMC was capable of increasing p65 protein levels in the nucleus. These results aligned with previous observations that α-MMC triggered the degradation of IκBα and therefore leads to the p65 nuclear translocation.

Next, we further determined whether the α-MMC-induced inflammatory responses could be inhibited by the pretreatment of specific IKKβ inhibitors. Treatment of cells with specific IKKβ inhibitors TPCA-1, SC-514, or BMS-345541 suppressed the α-MMC-induced IL-1β protein expression (Figure 3B). Furthermore, IKKβ inhibitor TPCA-1 was shown to inhibit α-MMC-induced p65 nuclear translocation (Figure 3C), cytokine secretion (Figure 3D), and cytokine mRNA levels (Figure 3E). Overall, these results suggested that IKK-NF-κB activation is indispensable for α-MMC inflammatory action.

### 2.5. α-MMC-Induced Inflammatory Responses via JNK Pathway

**Figure 4:**
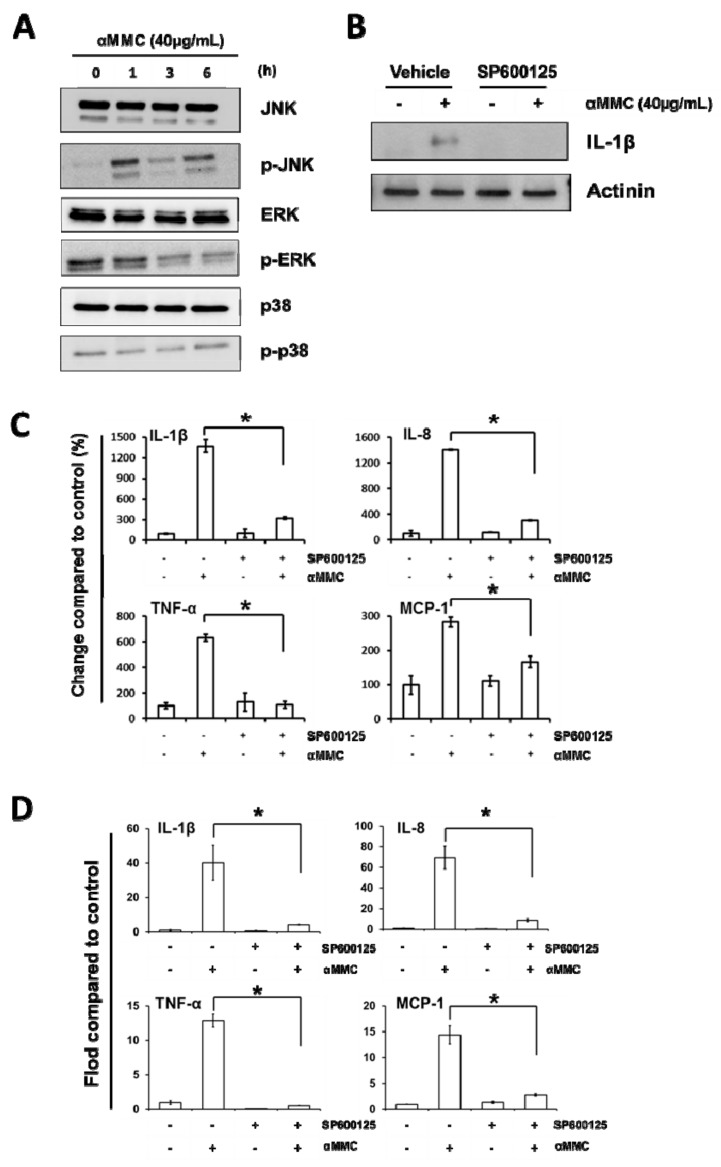
JNK activation is essential in α-MMC-induced inflammatory responses in THP-1 cells. (**A**) Western blot of THP-1 cells incubated with α-MMC (40 μg/mL) for different time intervals (0, 1, 3, 6 h). Whole-cell extracts were extracted and phosphorylated JNK, ERK, and p38 levels were analyzed by Western blot analysis using corresponding antibodies. (**B**) THP-1 cells were firstly treated with 15 μM JNK inhibitor SP600125 for 30 min, followed by adding of α-MMC (40 μg/mL) and further incubated for 24 h. Whole cell lysates were extracted and protein expression levels of IL-1β were analyzed by Western blot. Actinin was employed as an equal loading control. (**C**) THP-1 cells were exposed to vehicle or α-MMC (40 μg/mL) for 24 h after pretreatments with or without 15 μM JNK inhibitor SP600125 for 30 min. Secretions of IL-1β, IL-8, TNF-α, and MCP-1 in THP-1 cells culturing medium were detected by ELISA. The results are shown as a percentage of change compared to control group. Data represent the mean ± SD of three replicates. Significant differences: * *p* < 0.05. (**D**) THP-1 cells were incubated with 15 μM SP600125 for 30 min prior to the treatment of 24 h α-MMC (40 μg/mL). The mRNA levels of the indicated cytokines were analyzed by RT-PCR. Results are shown as fold-change compared to the control. Data represent the mean ± SD of three replicates. Significant differences: * *p* < 0.01.

Previous reports showed that inflammatory responses of RIPs can be triggered by activation of MAPK kinases including JNK, p38, and ERK [12,29,30]. To determine whether α-MMC would induce the MAPK kinase pathway, the JNK, p38, and ERK activities of α-MMC-treated THP-1 cells were assayed using Western blot analysis. We detected elevated levels of phosphorylated JNK, but not phosphorylated p38 or ERK, in THP-1 cells incubated with α-MMC (Figure 4A). This observation matches with previous results that α-MMC was able to activate the JNK pathway in liver cells [31]. To determine whether cytokine expression induced by α-MMC is dependent on the JNK pathway, we pre-treated THP-1 cells with JNK inhibitor SP600125 for 30 min before the addition of α-MMC. Treatment of JNK inhibitor SP600125 was shown to inhibit α-MMC-induced IL-1β protein expression (Figure 4B), cytokine secretion (Figure 4C), and cytokine mRNA levels (Figure 4D). Overall, these results indicated that activation of JNK pathway is essential for α-MMC-induced inflammatory responses.

### 2.6. Secretion of IL-1β, TNF-α, and MCP-1 in the Serum of α-MMC-Administrated Mice

**Figure 5:**
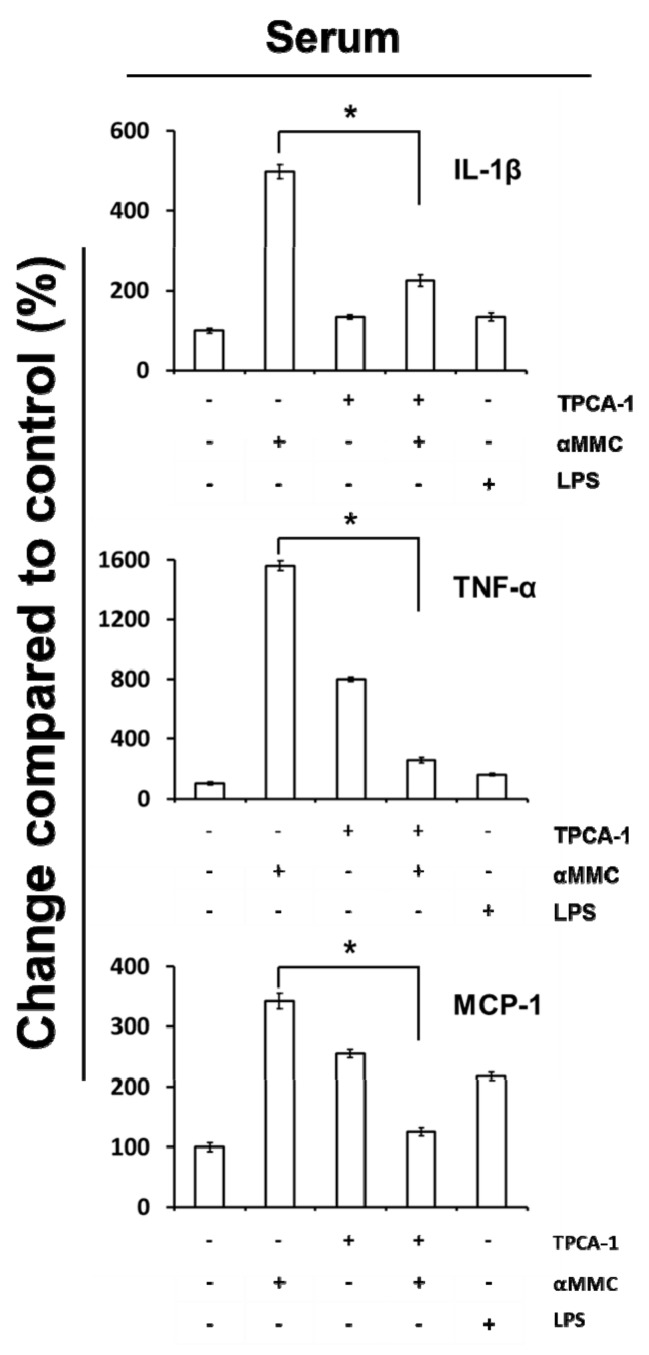
Inflammatory responses in mice administration with α-MMC and α-MMC plus TPCA-1. ICR mice were treated with α-MMC (6 mg/kg), IKKβ inhibitor TPCA-1 (15 mg/kg), or their combination for three constitutive days. LPS (10 mg/kg) was used as positive control. Levels of IL-1β, TNF-α, and MCP-1 in the sera were detected by ELISA. The data present the mean ± S.E.M., *n* = 4. Significant differences: * *p* < 0.05.

We next determined if the treatment of α-MMC to mice would promote the induction of cytokines in serum. Mice with groups of four treated with vehicle control, TPCA-1 alone, α-MMC alone, and TPCA-1 combined with α-MMC were exsanguinated finally after drug administration. IL-1β, TNF-α, and MCP-1 protein levels in serum were analyzed by mouse cytokine ELISA kits (eBioscience, San Diego, CA, USA). We observed that the levels of IL-1β, TNF-α, and MCP-1 proteins in serum were upregulated upon α-MMC treatment and these effects were inhibited by co-administration of IKKβ inhibitor TPCA-1 (Figure 5). In summary, these data suggested that the α-MMC would induce inflammatory responses in vivo and these effects were dependent on NF-κB activation.

## 3. Discussion

There were a few reports indicated the immune-modulating action of *Momordica charantia* [19,20,21]. In this study, we determine that α-MMC induces inflammatory cytokines secretion in human monocytes via NF-κB and JNK pathways. These findings indicate that the immunomodulative properties of *Momordica charantia* may be triggered by α-MMC present in the *Momordica charantia* extract.

In current study, the pro-inflammatory effects of α-MMC were investigated by expressing recombinant proteins in bacteria rather than isolating the native plant-purified proteins. In most studies, RIPs are isolated from plants using a chromatological method. However, there are numerous inbuilt problems emerging during the isolation of native RIPs, like insufficient source materials and complex purification processes. Therefore, production of RIPs using recombinant techniques may be a good way to solve these manufacturing problems. Moreover, recombinant RIPs can gain further advantages for cell penetration and increased plasma half-life, either by fusion with a chemical linker or with antibodies or other applicable carriers, when compared with native RIPs [1]. However, the critical issue regarding this technique is whether the recombinant protein will possess the same activity compared with the native protein. A previous study reported that recombinant RIP expressed in *E. coli* might be inactive due to the lack of post-translational modification [32]. In the current study, the heterologous expression and purification steps of α-MMC were performed according to experimental procedures described by Ding’s research group [33]. The recombinant α-MMC produced by this expression and purification protocol has been shown to exert *N*-glycosidase, DNA-nuclease, antibacterial, and also antifungal activities [33,34,35]. In our study, we revealed that there was no apparent difference between the cytotoxicity triggered by native and recombinant forms of α-MMC [31,36,37]. Upon α-MMC treatment, the cytotoxic effects were comparable between the native α-MMC [31,36,37] and recombinant α-MMC used in the current study (Figure 1C). Moreover, we showed that recombinant α-MMC used in this study is active from its DNA nuclease activity (Figure S6). However, in the current study we cannot conclude whether the native and recombinant α-MMC proteins share identical cellular activities. At this point we do not know whether the purified recombinant α-MMC used in this study would exert RNA hydrolase activity, rRNA N-glycosidases activity, and protein synthesis inhibitory activity as reported [21,22,23,24], and also the relationship between these activities and its inflammatory inducing properties is still not clear. In addition to this, a previous study demonstrated that α-MMC could get into cells through the LRP1 mediated endocytosis pathway [31]. Therefore, further studies on the enzymatic activity, protein translation inhibitory abilities, and the cell entry mechanism must be carried out as described [38] to clarify the differences between native or recombinant α-MMC and also the inflammatory inducing mechanism and activity of recombinant α-MMC in the future.

We carried out the inflammatory responses and cytokine production experiments with α-MMC concentration at 40 µg/mL (about 1.3 µM) that produced minor toxicity to cells (Figure 1C). Further investigation is needed to determine whether this dose can be reachable in the bloodstream in vivo or not. The average adult body contains around 5 L of blood and therefore 200 mg of α-MMC has to be taken to achieve the concentration 40 µg/mL, with the assumption that there is no gastric degradation of α-MMC. Based on the literature, 220 mg of native α-MMC protein could be collected by extracting 200 g of the seeds of *Momordica charantia* [20]. Therefore, α-MMC concentration in blood is depended on the uptake amount of bitter melon seeds. Moreover, the half-life of RIPs in plasma is comparatively short. For instance, trichosanthin, a RIP from *Trichosanthes kirilowii*, gives a plasma half-life of 8–12 min in animals [39]. Persistent consumption of bitter melon seeds is therefore required to sustain an immune-inducing dose in the body. Given that bitter melon is commonly employed as a granule and powdered nutritional supplement by Chinese medical doctors and the public for relieving high blood pressure, high cholesterol level, and lowering the blood glucose, more investigation is required to investigate the chronic pro-inflammatory effects of α-MMC for its safety use.

Previous studies showed that RIPs or RIP-containing plant extracts could induce cytokine production in both human and animals. The routes of administration of RIPs or RIP-containing plant extracts in animal experiments include intravenously [15], subcutaneously [40], and intraperitoneally injection [8,14], indicating that the immuno-inducing properties of RIPs or RIP-containing plant extracts were not affected by different administration methods. Meng et al. revealed that the SD rats body weight treated by 6.25 mg/kg of α-MMC dropped remarkably [20]. Our data showed that administration of 6 mg/kg did not affect the weights of mice (unpublished data). Therefore, in this study, we administered α-MMC at 6 mg/kg for three constitutive days in order to test the immuno-inducing effects.

The present data suggest a new and distinct model of why consumption of *Momordica charantia* may contribute to inflammatory-related disease, due to the facts that α-MMC could induce inflammatory responses via upregulating cytokines’ expression in immune cells. In our study, α-MMC was found to increase MCP-1, TNF-α, IL-8, and IL-1β and mRNA levels, as well as these secreted proteins, in THP-1 monocytic cells (Figure 2). The results presented in this paper also demonstrated the function of IKK/NF-κB and JNK activations in the initiation of α-MMC-mediated inflammatory responses (Figure 3 and Figure 4). Despite the fact that our results are promising, more effort is required to explain how JNK and IKK are being activated by α-MMC. Previous results showed that JNK and IKK pathways can be activated by the TAK1 pathway [41]. Further studies using anti-TNF and IL-1b antibodies and TAK1/JNK/IKK depletion are needed to investigate the role of these pathways in inflammatory pathways triggered by α-MMC. Moreover, the functional outcome of the α-MMC-stimulated immune cells is still not clear. The immune system works as defense system within humans to protect against disease. Thus, additional studies on the role of α-MMC in the immune system are needed in future. Given that α-MMC and other RIPs were observed to exert anti-tumor effects in various types of cancer cells [31,36,37,42], however, their inflammation inducing properties may hamper its potential use as a cancer therapeutic agent. Thus, the development of NF-κB and JNK inhibitors are certainly obvious candidates as combinatorial agents for α-MMC intoxication to deal with its proinflammatory effects. Moreover, based on its therapeutic potential in clinic, the anticancer outcome of cotreatment of α-MMC and NF-κB/JNK inhibitors will be worth further investigation in different cancer cell lines. In this research, we confirmed the role of JNK and IKK/NF-kB pathways in the MMC-induced inflammatory responses which provides additional mechanistic data to the similar observation made by Deng et al. [43]. On the other hand, we observed different α-MMC-regulated inflammatory effects in compared with Deng et al. findings [44], in which a sub-lethal concentration (80 μg/mL) of α-MMC inhibited cytokines via JNK activation, leading to the decrease of immuno-response in cells. However, in our results, we found that a comparatively lower dose of α-MMC (40 μg/mL) would induce inflammatory cytokine expression through IKK/NF-κB and JNK activation. Further efforts are needed to study the differential dosage responses triggered by α-MMC.

## 4. Conclusions

The present study demonstrates that alpha-momorcharin (α-MMC) triggers pro-inflammatory responses via promoting NF-κB and JNK activities in cell culture monocytes and a mouse model. α-MMC is a RIP found in powder form in the seeds of an edible plant *Momordica charantia* and it is actually eaten raw. Therefore, further work is needed to study the detailed inflammatory and pharmacokinetic properties of α-MMC and its linkage to the side effects of the seed of *Momordica charantia*, in order to establish a safety guideline for the consumption of these RIP-containing edible plants.

## 5. Materials and Methods

### 5.1. Materials

The anti-His-tagged, anti-IκBα, anti-IL-1β, anti-PCNA, MAPK Family Antibody Sampler Kit and Phospho-MAPK Family Antibody Sampler Kit were supplied by Cell Signaling Technology (Beverly, MA, USA). Antibodies against p65 and actinin were obtained from Santa Cruz Biotechnology (Santa Cruz, CA, USA). BMS-345541, TPCA-1, SC-514, and SP600125 were purchased from Selleckchem. 3-(4,5-Dimethyl-2-thiazolyl)-2,5-diphenyl-2H-tetrazolium bromide (MTT), Isopropyl β-d-1-thiogalactopyranoside (IPTG) and imidazole were purchased from Sigma Chemical Co. (St. Louis, MO, USA). Human Inflammatory Response and Autoimmunity RT^2^ Profiler™ PCR Array (PAHS-077ZA-6) was purchased from Qiagen (Valencia, CA, USA). All human and mouse cytokines ELISA kits were obtained from eBioscience (San Diego, CA, USA). Zeba™ spin desalting columns, HisPur Ni-NTA superflow agarose, high-capacity endotoxin removal spin columns and LAL chromogenic endotoxin quantitation kit were purchased from Thermo Scientific (Rockford, IL, USA). Rosetta™ 2 (DE3) pLysS competent cells were obtained from EMD Millipore Corporation (Billerica, MA, USA).

### 5.2. Expression and Purification of Recombinant α-MMC

The expression and purification of α-MMC was carried out as reported by Wang et al. [33] with slight modification. Briefly, we cloned the cDNA of α-MMC recaptured from the NCBI data bank (GenBank: X57682.1) into pET28a(+) vector and then followed by transformation into Rosetta (DE3) pLysS competent cells. Recombinant α-MMC protein was produced by adding IPTG (0.1 mM) for 16 h at 37 °C. Cells were collected and sonicated in phosphate-buffered saline (PBS) (pH 7.4), and the supernatant was put to HisPur Ni-NTA superflow agarose, followed by washing with binding/washing buffer (20 mM phosphate-buffered saline, 0.5 M sodium chloride, pH 6.0), the agarose was eluted by a graduate increase of imidazole concentration (10–200 mM). Recombinant α-MMC solutions were passed twice through Zeba™ spin desalting columns and endotoxin withdrawing centrifuge columns (high capacity) to remove imidazole as well as enotoxin residues, respectively. Endotoxin contamination was kept below the detection limit (0.1 EU/mL) of LAL chromogenic quantitation kit (Thermo Scientific). The concentration and the purity of α-MMC recombinant protein were analyzed by 12% SDS polyacrylamide gel electrophoresis using BSA as the benchmark.

### 5.3. Cell Cultures

Human monocytic THP-1 cells obtained from ATCC (Manassas, VA, USA) and cultured in RPMI medium (Invitrogen, Carlsbad, CA, USA) accompanied by 5% FCS, penicillin and streptomycin. Cells were maintained at 37 °C at 5% CO_2_ and 95% humidity.

### 5.4. Cytotoxicity Assay

Cytotoxicity was verified by the cytotoxicity assay as described previously [45]. In brief, 1 × 10^4^ THP-1 cells were seeded in 96-well plates and then untreated or treated with different doses of α-MMC (0–160 μg/mL) for 24 h. Then, cells were incubated with MTT reagent (5 mg/mL) at 37 °C for 4 h. Afterward, medium was discarded and MTT crystals were dissolved in 100 µL of DMSO. The optical densities (570 nm) were measured by spectrophotometer. The sublethal dose is defined as the dose leading to growth inhibitory effect below 20% (<IC20).

### 5.5. Enzyme-Linked Immunosorbent Assay (ELISA)

THP-1 cells were seeded in Opti-MEM in 24-well plates and then treated with vehicle or α-MMC (40 μg/mL) at 37 °C for 24 h. Cell culture supernatants were isolated and the cytokines concentrations were determined by cytokine ELISA Assay Kit (for human, eBioscience) according to the manufacturer’s instructions. Cytokines levels in cell medium were normalized and shown as pg/mL/mg of protein and the corresponding protein levels of cellular extracts were determined by Bradford assay. The cytokines concentrations in mouse serum were determined by mouse ELISA Assay Kit (eBioscience) following the manufacturer’s guideline.

### 5.6. Reverse Transcription-PCR and Real-Time PCR (RT-PCR) Analysis

RT-PCR analysis was performed as mentioned previously [46]. Briefly, THP-1 cells were incubated with vehicle control or α-MMC (40 μg/mL) for 24 h at 37 °C and the total RNA was then extracted using TRIzol assay (Invitrogen), followed by conducting the reverse transcription using the Prime Script RT Kit (Takara, Shiga, Japan) according to the manufacturer’s protocol. cDNA products were analyzed by Human Inflammatory Response and Autoimmunity RT^2^ Profiler™ PCR Array following the manufacturer’s instructions. LPS at sub-lethal dose of 1 µg/mL was used as positive control. Alternatively, real-time PCR on cDNA was carried out in an Applied Biosystems ViiA 7 real-time PCR machine (Thermo Fisher Scientific, Carlsbad, CA, USA) using SYBR Green assays (Bio-Rad, Hercules, CA, USA). The PCR primers were as follows: 5′-TTCGACACATGGGATAACGAGG-3′ and 5′-TTTTTGCTGTGAGTCCCGGAG-3′ for human IL-1β; 5′-ACTGAGAGTGATTGAGAGTGGAC-3′ and 5′-AACCCTCTGCACCCAGTTTTC-3′ for human IL-8; 5′-GAGGCCAAGCCCTGGTATG-3′ and 5′-CGGGCCGATTGATCTCAGC-3′ for human TNF-α; 5′-CAGCCAGATGCAATCAATGCC-3′ and 5′-TGGAATCCTGAACCCACTTCT-3′ for human MCP-1.

### 5.7. Western Blot Analysis

THP-1 cells were incubated with α-MMC (40 μg/mL) for indicated time intervals and the level of ERK, p-ERK, IκBα, p65, IL-1β, JNK, p-JNK, p38, p-p38 proteins in the whole-cell lysates and p65 protein in the nuclei were assayed by Western blotting as previously described [46]. Densitometric analysis of Western blots is shown in Supplementary Materials (Figures S1–S5).

### 5.8. Animal Treatments

Male ICR mice were obtained from the Chinese University of Hong Kong and kept under normal condition. Mice were randomly assigned to five groups (*n* = 4) and administered with saline, LPS (10 mg/kg, positive control), α-MMC (6 mg/kg), TPCA-1 (15 mg/kg), or α-MMC/TPCA-1 combination intragastrically (i.g.) for three constitutive days. At the end of treatment, retro-orbital blood samplings under anesthesia were performed and blood was collected. Blood samples were then centrifuged for 10 min at 2000× *g*. Then, serum was isolated, aliquoted and kept at −80 °C. The cytokines’ concentrations in mouse serum were determined by mouse ELISA Kit (eBioscience). The animal experiment was performed under the animal guideline of Hong Kong Baptist University (Permission number: HASC/12-13/0010, Date of approval: 01-04-2013) and Declaration of Helsinki and the Guide for the Care and Use of Laboratory Animals (the US National Institutes of Health). All animal experiments were performed under the principles of the 3Rs (replacement, reduction, and refinement).

### 5.9. Statistical Analysis

Software Origin 6.0 was employed for analyzing the statistical data (Origin, Northampton, MA, USA). Data are shown as mean ± standard deviation of three independent studies. Unpaired Student’s *t* tests were carried out and *p*-values less than 0.05 were considered to be statistically significant.

## Figures and Tables

**Table 1:** Profiling of recombinant α-MMC-induced inflammatory responses using inflammatory and autoimmunity PCR Array. THP-1 cells were treated with vehicle control, α-MMC (40 μg/mL) or LPS (1 μg/mL) for 24 h. Results are shown as the fold-change compared to control group. The upregulated genes with fold change >2 are shown. N/A = fold change <2. Results are representative of two independent experiments.

Symbol	Upregulated Gene Expression		
	Gene Name	Fold Change (>2)	
		αMMC	LPS
BCL6	B-cell CLL/lymphoma 6	4.42	8.02
C3	complement C3	13.58	15.79
C3AR1	complement C3a receptor 1	6.58	14.09
CCL11	C-C motif chemokine ligand 11	N/A	3.91
CCL13	C-C motif chemokine ligand 13	14.79	35.68
CCL16	C-C motif chemokine ligand 16	N/A	4.73
CCL19	C-C motif chemokine ligand 19	N/A	8.44
CCL2	C-C motif chemokine ligand 2	79.86	435.64
CCL3	C-C motif chemokine ligand 3	36.62	188.18
CCL4	C-C motif chemokine ligand 4	112.39	806.76
CCL5	C-C motif chemokine ligand 5	N/A	2.78
CCL7	C-C motif chemokine ligand 7	9.93	18.16
CCL8	C-C motif chemokine ligand 8	8.58	23.23
CCR4	C-C motif chemokine receptor 4	N/A	2.99
CCR7	C-C motif chemokine receptor 7	11.47	106.82
CD14	CD14 molecule	3.98	22.67
CD40	CD40 molecule	8.89	9.11
CSF1	colony stimulating factor 1	N/A	2.09
CXCL1	C-X-C motif chemokine ligand 1	27.92	55.1
CXCL10	C-X-C motif chemokine ligand 10	100.52	172.33
CXCL2	C-X-C motif chemokine ligand 2	29.89	57.36
CXCL3	C-X-C motif chemokine ligand 3	17.37	24.9
CXCL6	C-X-C motif chemokine ligand 6	6.41	9.84
CXCL9	C-X-C motif chemokine ligand 9	14.82	76.48
FASLG	Fas ligand	3.32	N/A
IFNG	interferon gamma	5.77	2.45
IL10	interleukin 10	N/A	7.81
IL15	interleukin 15	2.74	5.62
IL17A	interleukin 17A	N/A	2.45
IL1A	interleukin 1 alpha	4.14	5.8
IL1B	interleukin 1 beta	60.4	314.3
IL1R1	interleukin 1 receptor type 1	N/A	2.76
IL1RN	interleukin 1 receptor antagonist	3.37	8.5
IL22	interleukin 22	3.18	5.36
IL23A	interleukin 23 subunit alpha	8.46	39.64
IL6	interleukin 6	187.71	3140.98
CXCL8	C-X-C motif chemokine ligand 8	135.62	186.63
IL9	interleukin 9	N/A	4.19
LTA	lymphotoxin alpha	7.14	3.55
LTB	lymphotoxin beta	2.57	6.95
LY96	lymphocyte antigen 96	5.56	13.53
MYD88	myeloid differentiation primary response 88	2.79	3.14
NFKB1	nuclear factor kappa B subunit 1	4.65	4.14
PTGS2	prostaglandin-endoperoxide synthase 2	3.06	5.77
RIPK2	receptor interacting serine/threonine kinase 2	N/A	2.01
SELE	selectin E	N/A	8.25
TLR1	toll like receptor 1	5.38	5.75
TLR3	toll like receptor 3	4.24	8.76
TLR6	toll like receptor 6	3.81	3.34
TLR7	toll like receptor 7	4.6	6.17
TNF	tumor necrosis factor	3.73	12.58

## Supplementary Materials

Information below is available online at https://www.mdpi.com/2072-6651/11/12/694/s1, Figure S1: Densitometry values for Figure 3A. Densitometry data for the IkBα and p65 (with normalization to actinin or PCNA and their vehicle control) are presented below every lane, Figure S2: Densitometry values for Figure 3B. Densitometry data of IL-1β (with normalization to actinin their vehicle control) are presented below every lane, Figure S3: Densitometry values for Figure 3C. Densitometry values for the p65 levels (with normalization to PCNA and the corresponding vehicle control) are presented below every lane, Figure S4: Densitometry values for Figure 4A. Densitometry data of the p-JNK, p-38 and p-ERK levels (with normalization to JNK, p38 and ERK respectively and their corresponding vehicle control) are presented below every lane, Figure S5: Densitometry values for Figure 4B. Densitometry data of IL-1β (with normalization to actinin their corresponding vehicle control) are presented below every lane. Figure S6: DNA-nuclease activity of α-MMC towards plasmid pCMV6. DNA-nuclease activity analysis was performed according to method described previously [33] and the plasmid cleaving effect was investigated by DNA gel electrophoresis with GelRed. Plasmid pCMV6 was degraded when it was co-incubated with increasing concentrations of α-MMC (from 0 to 4 μg). To further confirm that the role of Mg2+ ions in the DNA cleaving effect, EDTA containing assay reagents were employed. The cleaving effect of α-MMC was rescued under EDTA treatment, indicating that magnesium metal had an essential role in the α-MMC-induced DNA cleaving effect.
