# Supplementary material for: Ribosome-Inactivating Protein α-Momorcharin Derived from Edible Plant Momordica charantia Induces Inflammatory Responses by Activating the NF-kappaB and JNK Pathways
Source: Toxins (Basel). 2019 Nov 26;11(12):694. doi: 10.3390/toxins11120694 (PMC6949964; doi:10.3390/toxins11120694)
Supplement: Supplementary file 1 [file toxins-11-00694-s001.pdf]

Supplementary Materials: Ribosome-Inactivating Protein α-Momorcharin Derived from Edible Plant *Momordica charantia* Induces Inflammatory Responses by Activating the NF-kappaB and JNK Pathways

Ying-Jie Chen, Jia-Qian Zhu, Xiu-Qiong Fu, Tao Su, Ting Li, Hui Guo, Pei-Li Zhu, Sally Kin-Wah Lee, Hua Yu, Anfernee Kai-Wing Tse and Zhi-Ling Yu

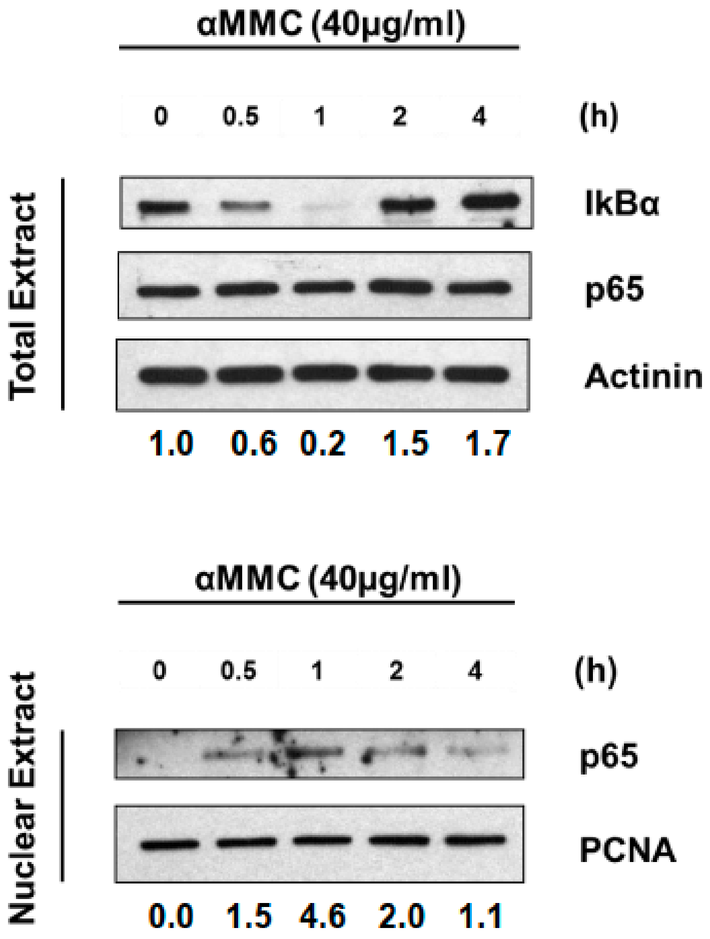

Figure S1. Densitometry values for Figure 3A. Densitometry data for the IkBα and p65 (with normalization to actinin or PCNA and their vehicle control) are presented below every lane.

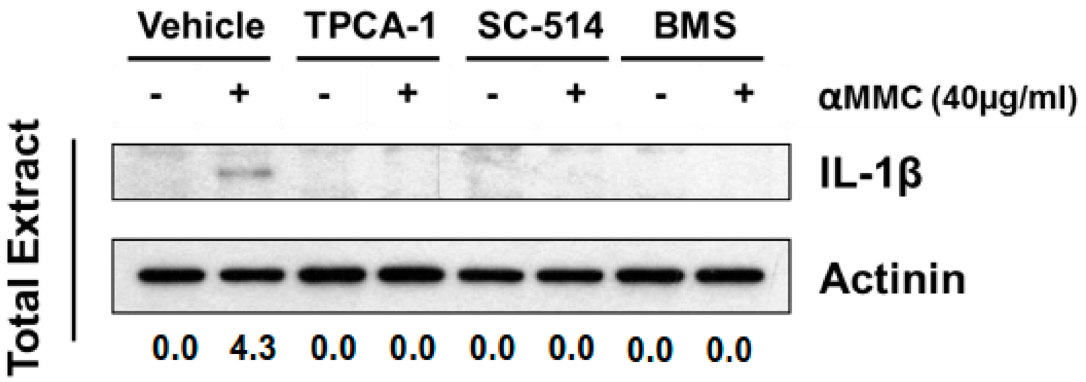

Figure S2. Densitometry values for Figure 3B. Densitometry data of IL-1β (with normalization to actinin their vehicle control) are presented below every lane.

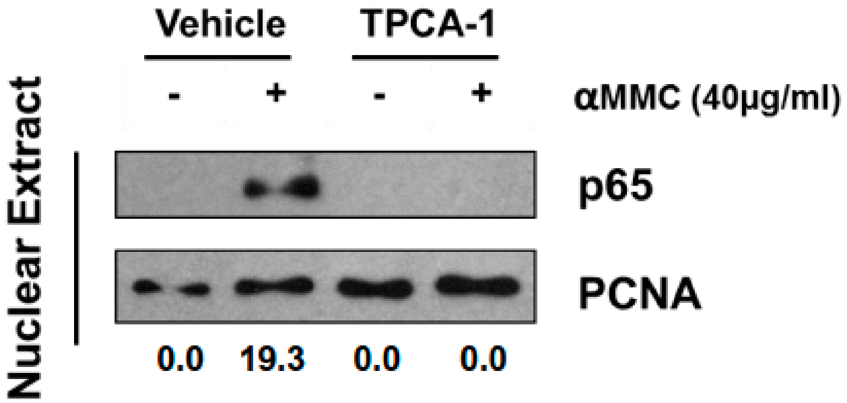

Figure S3. Densitometry values for Figure 3C. Densitometry values for the p65 levels (with normalization to PCNA and the corresponding vehicle control) are presented below every lane.

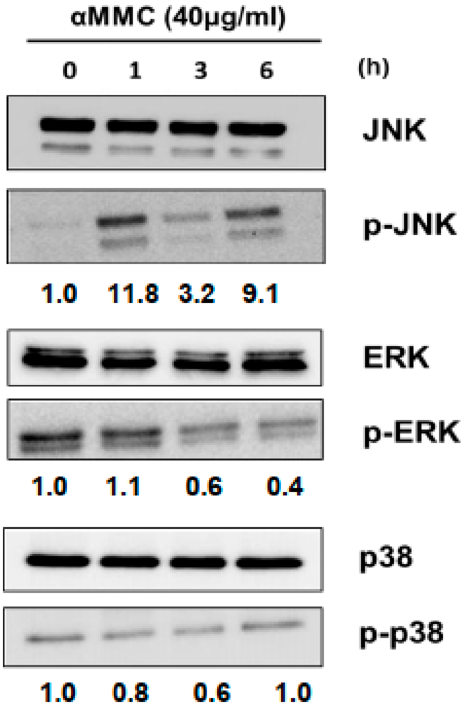

Figure S4. Densitometry values for Figure 4A. Densitometry data of the p-JNK, p-38 and p-ERK levels (with normalization to JNK, p38 and ERK respectively and their corresponding vehicle control) are presented below every lane.

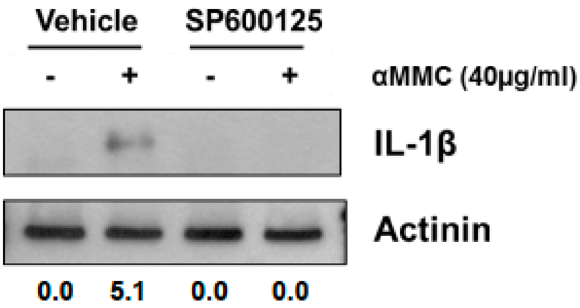

Figure S5. Densitometry values for Figure 4B. Densitometry data of IL-1β (with normalization to actinin their corresponding vehicle control) are presented below every lane.

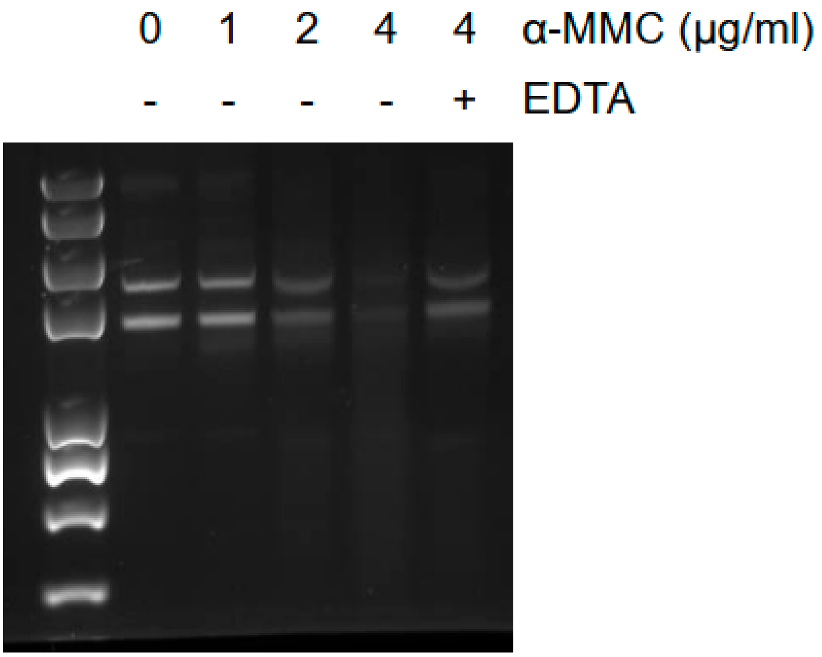

Figure S6. activity of α-MMC towards plasmid pCMV6. DNA-nuclease activity analysis was performed according to method described previously [33] and the plasmid cleaving effect was investigated by DNA gel electrophoresis with GelRed. Plasmid pCMV6 was degraded when it was co-incubated with increasing concentrations of α-MMC (from zero to four μg). To further confirm that the role of Mg2+ ions in the DNA cleaving effect, EDTA containing assay reagents were employed. The cleaving effect of α-MMC was rescued under EDTA treatment, indicating that magnesium metal had an essential role in the α-MMC-induced DNA cleaving effect.
